# The Association Between Parents’ Physical Function and Adult Children’s Caregiving Burden

**DOI:** 10.1093/geroni/igab046.769

**Published:** 2021-12-17

**Authors:** Stephanie Bergren, Qun Le, Dexia Kong, XinQi Dong

**Affiliations:** 1 Rutgers University, New Brunswick, New Jersey, United States; 2 The Chinese University of Hong Kong, Hong Kong , Hong Kong; 3 Rutgers University, Rutgers Institute for Health, New Jersey, United States

## Abstract

Using data from 544 older parents-adult children Chinese American dyads, this study aims to understand the association between older parents’ physical function and their adult children’s perceived caregiving burden. Parents’ physical function was assessed by the Katz Index of Activities of Daily Living (ADL) and the Lawton Instrumental ADL (IADL), with higher scores indicating more functional limitations. Adult children’s caregiving burden was assessed in five dimensions, including time dependence, developmental, physical, social, and emotion burden. Logistic regression was used to examine the association. More ADL limitations were associated with a higher likelihood of developmental burden (OR:1.14 (1.06-1.23)) and physical burden (OR:1.14 (1.06-1.23)) burden. More IADL limitations was associated with a higher likelihood of time dependence burden (OR:1.08 (1.03-1.12)), developmental burden (OR:1.06 (1.03-1.09)), and physical burden (OR:1.08 (1.04-1.12)). Parents’ physical function was not related to children’s social and emotional burdens. Practice and research implications will be discussed.

